# Epigallocatechin Gallate Enhances Inhibition Effect of DDP on the Proliferation of Gastric Cancer BGC-823 Cells by Regulating p19^Arf^-p53-p21^Cip1^ Signaling Pathway

**DOI:** 10.31557/APJCP.2021.22.4.1263

**Published:** 2021-04

**Authors:** Mengya Xue, XiaoLv Liu, Bing Cheng, XingKai Rui, MingCai Wu, Jun Lv

**Affiliations:** 1 *Department of Biochemistry and Molecular Biology, Wannan Medical College, Wuhu, China. *; 2 *Wuhu Sub-center of Anhui International Travel Health Care Center, Wuhu, China. *

**Keywords:** EGCG, DDP, p19^Arf^-p53-p21^Cip1^ signaling pathway, BGC, 823 cells

## Abstract

**Objective:**

To indicate the effect of Epigallocatechin gallate (EGCG) and Cisplatin (DDP) on proliferation of gastric cancer BGC-823 cells and the relative underlying mechanism.

**Methods:**

Cultured BGC-823 cells were treated by 5 μg/mL DDP, 25 μg/mL EGCG and combined 5 μg/mL DDP with 25 μg/mL EGCG, a blank group was used as control. Cell morphology was observed by 4’,6-diamidino-2-phenylindole (DAPI) staining. The ability of cell proliferation was detected by 3-(4,5-dimethylthiazol-2-yl)-2,5-diphenyltetrazolium bromide (MTT)assay. The cell cloning rate was determined by colony formation assay. The ability of cell migration was detected by cell scratch test. The cell cycle distributions and apoptosis were analyzed by flow cytometry, The expression of *p19*^Arf^*, p53, p21*^Cip1^ mRNA was determined by RT-qPCR. The protein levels of p19^Arf^, p53, p21^Cip1^ were measured by Western blot.

**Results:**

Compared with DDP or EGCG treatment alone, EGCG combined with DDP treatment significantly caused nuclear shrinkage, reduced the proliferation rate, the ability of cell clone and migration. EGCG combined with DDP treatment caused cell cycle arrest in G1 phase in BGC-823 cells, increase of apoptosis (21.3%) vs EGCG (7.25%) and DDP (3.86%) single-use group (*P*<0.01), up-regulated gene and protein expressions of* p19*^Arf^*, p53, p21*^Cip1^ (*P*<0.01).

**Conclusion:**

EGCG can enhance the effect of DDP on inhibiting BGC-823 cell proliferation and inducing apoptosis via activating the p19^Arf^-p53-p21Cip1 signaling pathway.

## Introduction

As one of the malignant tumors that seriously endanger people’s health and life safety, gastric cancer has the characteristics of high incidence, rapid progress and poor prognosis (Iwasaki et al., 2020). Unhealthy eating habits, poor physical fitness, high pressure and other factors have led to the younger generation of gastric cancer. Although treatments such as surgery, radiotherapy and chemotherapy prolong the life of patients, the prognosis is poor and the 5-year survival rate is less than 10% (Zhao and Fu, 2019; Alshehri et al., 2020). Traditional chemotherapeutic drugs have been widely used in clinical therapeutic strategies while with the limitations of high toxic and side effects. For example, DDP is a metal chemotherapeutic drug, which inhibits DNA replication and thus affects cell growth and proliferation. However, its adverse reactions include gastrointestinal reactions, neurotoxicity, and allergic reactions, etc (Zou and Li, 2019; Liu and Wang, 2019). Low-dose DDP was used in anti-tumor therapy to reduce its side effects, but the effect of chemotherapy is reduced accordingly. Therefore, looking for the therapeutic strategy to improve the effect of low-dose cisplatin chemotherapy attracted wide interest of scientists, including the combination of cisplatin and naturally occurring components which have advantages of low toxicity and cost. 

Green tea, a natural drink, is a powerful antioxidant and a scavenger of free iron (Jakubczyk et al., 2020). The major polyphenolic compound with the strongest anti-tumor activity in green tea extract is EGCG, which can significantly inhibit malignant tumors with less toxic and side effects, and predict good prognosis of tumor patients (Du et al., 2012; Zhang et al., 2019; Zan et al., 2019; Tsai et al., 2016). Studies revealed that EGCG increased the protein expression of p53 in 3T3-L1 cells and then promoted cell apoptosis (Kumar et al., 2019). As Oya et al., (2017) reported in his previous work, EGCG could induce growth arrest and apoptosis of human lung cancer cells PC-9 through up-regulating the expression of p21^waf1^. Our previous experiments demonstrated that EGCG could up-regulate the expression of p19^Arf^,* p53*, and p21^Cip1 ^to induce apoptosis in acute myeloid leukemia cells, confirming that the p19^Arf^-p53-p21^Cip1 ^signaling pathway mediated EGCG-induced apoptosis (Wu et al., 2020).

Several studies have shown that EGCG can enhance the proliferation inhibitory effect of DDP on small cell lung cancer cells and colon cancer cells (Zhao et al., 2012; Gao and Sun, 2016). However, the effect and molecular mechanism of EGCG combined with DDP used in gastric cancer BGC-823 cells remained unclear. This study aimed to investigate the effect of the combination of EGCG and DDP on the proliferation and apoptosis of gastric cancer BGC-823 cells through in vitro experiments. Furthermore, we attempted to enucleate whether EGCG can enhance the proliferation inhibitory effect of cisplatin on gastric cancer cells by regulating the p19^Arf^-p53-p21^Cip1^ signaling pathway.

## Materials and Methods

*1.1 Materials and reagents*

Gastric cancer BGC-823 cells were purchased from Shanghai Cell Bank of the Type Culture Collection Committee of the Chinese Academy of Sciences. EGCG and DDP were obtained from Sigma, high-sugar DMEM medium and DAPI were purchased from Shanghai Sangon Biotech. Monoclonal anti-p19^Arf^, anti-p53, anti-p21^Cip1 ^and secondary antibodies were from Santa Cruz. Annexin V fluorescein isothiocyanate (FITC) kit and cell cycle detection kit were purchased from KeyGEN. SYBR Green qPCR Master Mix kit was obtained from Thermo. ECL luminescence kit was purchased from Shanghai Sangon Biotech.

*1.2 Methods*

*1.2.1 Cell culture*

DMEM medium supplemented with D-glucose and 10% fetal calf serum was used to culture human gastric cancer BGC-823 cells in an incubator in the atmosphere containing 5% CO_2_ with moderate humidity saturation, while the culture temperature was set at 37^o ^C.

*1.2.2 DAPI staining to observe cell morphology*

Dilute DAPI with PBS for later use, trypsinize logarithmic growth cells into single cells and start counting. After inoculating 96-well plates for adherent growth, add 5 µg/mL DDP, 25 µg/mL EGCG, DDP + EGCG (5 µg/mL, 25 µg/mL), after 24 hours of treatment, fix the cells, wash twice with PBS, stain for 5-20 min in the dark, rinse, and observe the cell morphology with a fluorescence microscope.

*1.2.3 MTT detects the inhibitory effect of EGCG and DDP on the proliferation of BGC-823 cells*

Digest the BGC-823 cells in the logarithmic growth phase and inoculate them in a 96-well culture plate. After culturing for 24 hours, add DDP (5 µg/mL), EGCG (25 µg/mL), DDP + EGCG (5 µg/mL DDP + 25 µg/mL EGCG). Each group had 5 parallel wells. After 24 hours, the culture medium was removed. After MTT treatment for 4 hours, the culture medium in the wells was carefully aspirated and Dimethyl sulfoxide (DMSO) was added to incubate for 10 minutes. After the MTT crystals in the well are completely dissolved and the color of the solution is measured on the microplate reader (Thermo) to determine the optical density value (A570 nm) of each group, the calculation formula is: cell proliferation rate = (experimental group A570 nm-zero adjustment group A570 nm)/(Control group A570 nm-zero adjustment group A570 nm)×100%.

*1.2.4 The cell cloning efficiency was analyzed by cell cloning formation assay (Kumar et al., 2019)*

BGC-823 cells was treated with drugs the same as in 1.2.2. The cells were diluted and slowly added and dispersed on the cell culture plate. After incubating for 12 d ~ 14 d, PBS was used to wash the cells twice, methanol was performed to fix the cells and 0.1% crystal violet was prepared to staine the cells. Then manually counte the visible colonies. The cloning efficiency was calculated with the following formula: The cloning efficiency (%) = (number of clones/number of cells inoculated) × 100%.

*1.2.5 The cell migration was detected by cell scratch test*

On the back of the culture plate, mark each well with at least 3 horizontal lines evenly with a marker pen, transfer the BGC-823 cells in the logarithmic growth phase to a 6-well cell plate for incubation, and add drugs after culturing for 24 hours (Same as 1.2.2), 3 parallel holes in each group, continuous culture for about 14 days. The cells grow to 90%, mark the scratches vertically, wash with PBS, change to serum-free medium and continue to incubate. The distance between cell scratches was calculated and recorded using microscope at 0 h and 24 h.

*1.2.6 The influence of EGCG and DDP on the cell apoptosis and cell cycle were analyzed by flow cytometry*

Trypsin the BGC-823 cells in logarithmic growth phase was digested and inoculated into a 6-well cell plate. The drug treatment same as 1.2.2 while the cells grow to 90% density, 5 µL AnnexinV-Fluorescein Isothiocyanate (AnnexinV-FITC) and 5 µL propidine iodide (PI) was added in sequence into 500 µL of cell suspension, shake and mix gently, and then keep it in the dark for 15 minutes. Cells were filtered by nylon omentum, and the apoptosis and cell cycle of BGC-823 cells were detected by flow cytometry.

*1.2.7 p19* ^Arf^ *, p53, p21* ^Cip1^ *mRNA expression were detected by RT-qPCR*

The drug treatment was the same as that in 1.2.2. The cells were harvested after incubating for 24 hours. Trizol reagent was used to extract the total RNA, and then was quantified by using NanoDrop micro spectrophotometer. cDNA was synthesized according to the instructions of PrimeScript RT Reagent Kit with gDNA Eraser (Takara). qPCR was performed according to the SYBR Green qPCR Master Mix (Thermo) operating instructions. The primers used were shown in Table 1. PCR conditions were: pre-denaturation at 95 ^o^C for 3 min, denaturation at 94 ^o^C, duration for 30 s, annealing temperatures were shown in Table 1, annealing time was 30 s and then extension at 72 ^o^C for 30 s. The reaction cycles was set as 35.The relative mRNA expression level was qualified using 2^−ΔΔCt^ method and β-actin was selected as the internal reference gene.

*1.2.8 p19* ^Arf^ *, p53, p21* ^Cip1^ *protein expression were determined by Western blot*

The cells after 24 hours of drug treatment as in 1.2.2 were collected to extract the total cellular protein, test the protein concentration was quantified using the bicinchoninic acid assay. 50 µg protein were subjected to 10% SDS-PAGE, and transferred to a polyvinylidene fluoride membrane. 5% skimmed milk was prepared to block the membrane for 1 hour, washed the membrane with TBST for 3 times. Then primary antibodies was diluted to incubate the membrane overnight at 4^o^C. Following the membrane was rinsed 3 times with TBST, subsequently, secondary antibodies were added and incubated for 2 hours with shaking slightly. ECL luminescent reagent was used to visualize the immuno-detected proteins, the Quantity One software version 4.6.2 was used to analyze the gray value.

## Results

*2.1 Cell morphology was visualized by DAPI staining*

**Figure 1:**
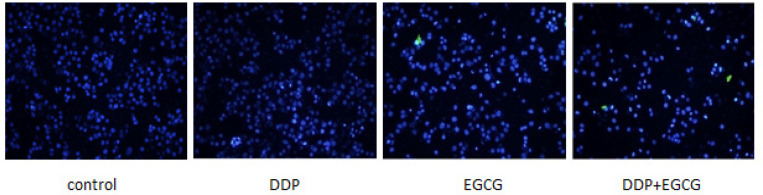
Cell Morphology was Visualized by DAPI Staining

As shown in Figure 1, cells staining with DAPI were excited blue-white fluorescencein the fluorescence microscope. The cellular nuclei treated with EGCG and DDP became concentrated while the nuclear staining is brighter and deeper, and the apoptotic cell ratio was higher than untreated group. The cells without drug treatment were slightly stained. As compared to individual treatments, the apoptotic cell ratio in co-treatment with EGCG and DDP was significantly increased.

*2.2 The effects of EGCG and DDP treatments on cell viability*

**Figure 2:**
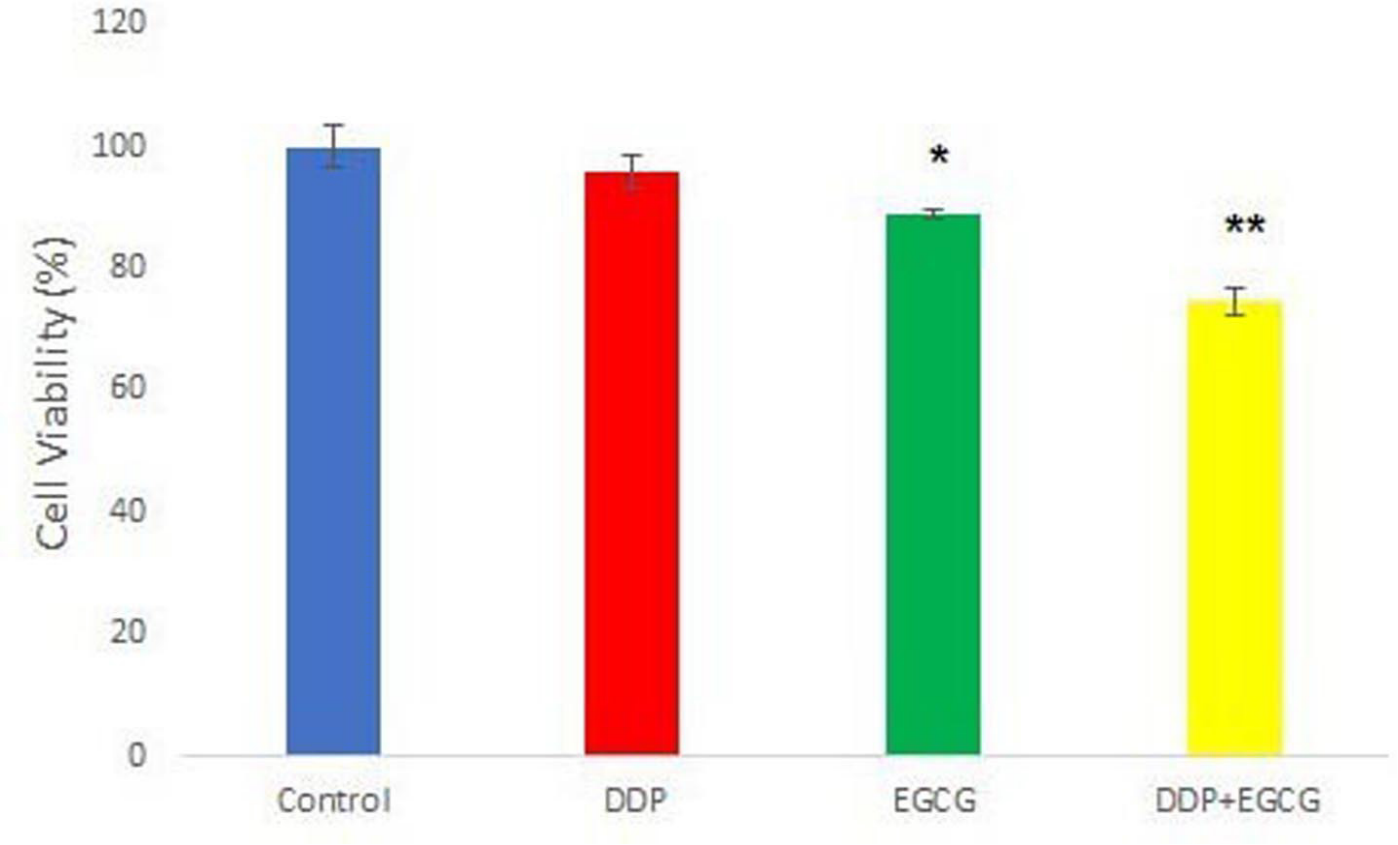
The Effects of EGCG and DDP Treatments on Cell Viability was Detected by MTT Assay. Compared with control * *P*<0.05,**P<0.01

The results revealed that co-treatment with EGCG and DDP or individual treatments inhibited the proliferation of gastric cancer cells BGC-823 as compared to the control group. Compared with the control cells, the cell viability in the combination of EGCG and DDP or treatment with either EGCG or DDP alone was significant decreased respectively (Figure 2). The co-treatment with EGCG and DDP caused greater decrease in BGC-823 cell viability as compared to that in cells treated with either EGCG or DDP alone, indicated that the combination of EGCG and DDP showed mutual promotion effects.

*2.3 Treatment of BGC-823 cells with EGCG and DDP inhibited the cloning efficiency*

Compared with the blank control cells, the cloning efficiency in cells with 5 µg/mL DDP and 25 µg/mL EGCG decreased. The colony formation assay showed that proliferation of EGCG combined with DDP treated cells decelerated significantly as compared with EGCG or DDP used alone, as shown in Table 2.

**Figure 3:**
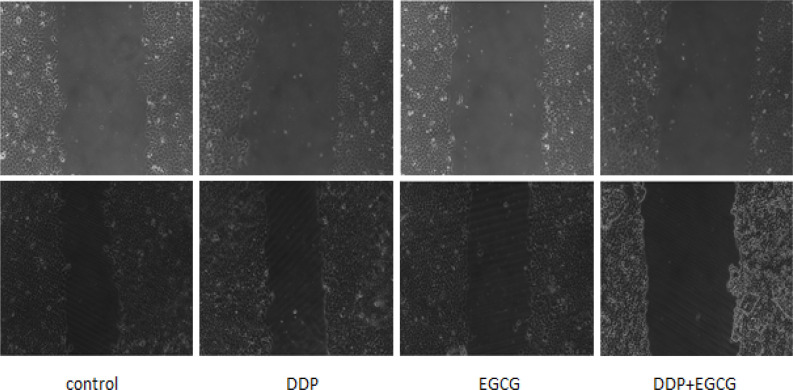
The Effects of EGCG and DDP Treatmentson the Cell Migration Ability(×100)

As shown in Figure 3, the distances between the scratches in cells of co-treatment with EGCG and DDP or individual treatments were wider than the control group after 24 hours of drug treatment, while co-treatment with EGCG and DDP caused the largest distance. The results revealed that co-treatment with EGCG and DDP or individual treatments inhibited cell migration, and the combination of EGCG and DDP exhibited significant inhibitory effect on BGC-823 cell migration ability.

*2.5 EGCG and DDP induces the apoptosis and influence the cell cycle of BGC-823 cells*

**Figure 4:**
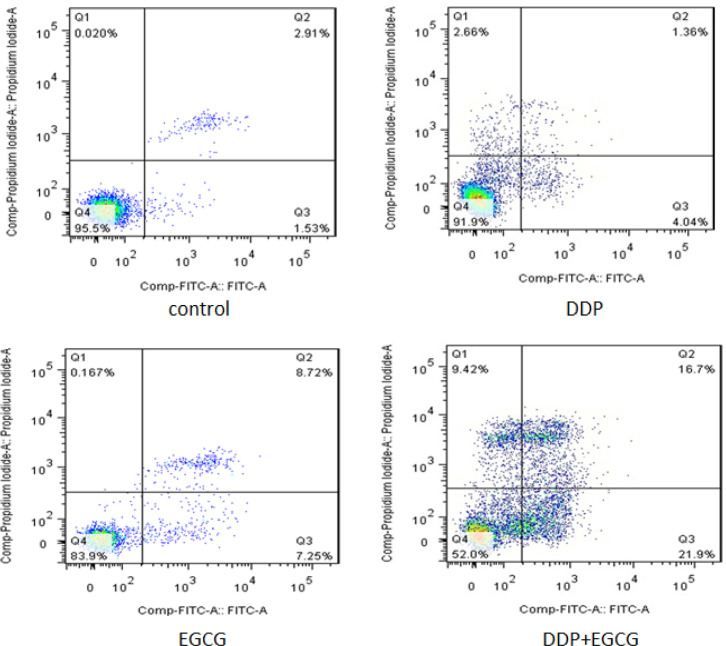
The Effects of EGCG and DDP Treatments on the Cell Apoptosis

**Figure 5:**
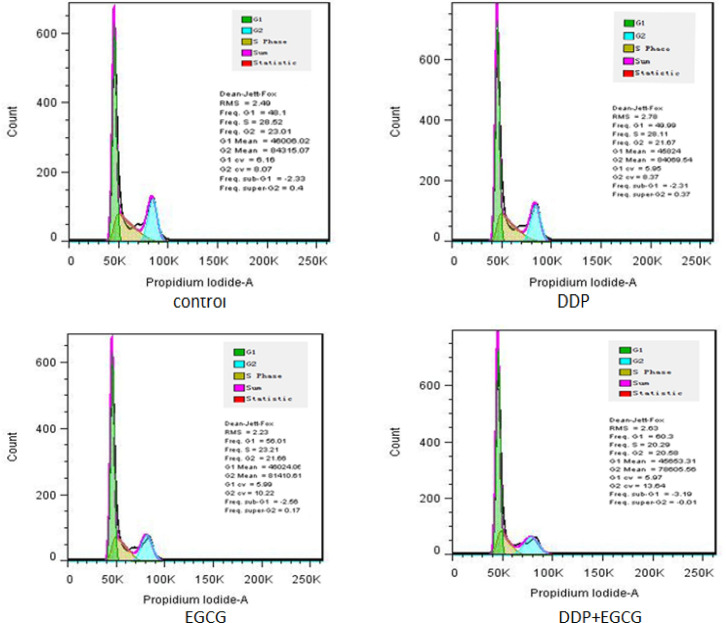
The Effects of EGCG and DDP Treatmentsonthe Cell Cycle of BGC-823 Cells

Compared with the control group, EGCG and DDP blocked the BGC-823 cells in G1 phase, caused increase of cells in S phase or G2 phase. Compared to the DDP-treated cells, EGCG increased the population of G0/G1, and decreased the population of cells in the S phase or G2 phase. Furthermore, co-treatment with EGCG and DDP significantly increased G1 population while the population of cells in the G2 and S phases was decreased compared with the individual treatments (Figure 4). Moreover, the effect of EGCG and DDP on BGC-823 cells apoptosis was evaluated.BGC-823 cells were stained with Annexin V and PI, the early apoptosis rate was detected by flow cytometry (Figure 5). The results revealed that the early apoptosis rate (21.3%) was notably increased by co-treatment with EGCG and DDP, which was significantly higher than that in cells treated with EGCG (7.25%) and DDP (4.04%) alone respcetively (*P*<0.01).

*2.6 Treatment of BGC-823 cells with EGCG and DDP increased p19* ^Arf^ *, p53, and p21* ^Cip1^ *mRNA and protein expression levels*

**Figure 6:**
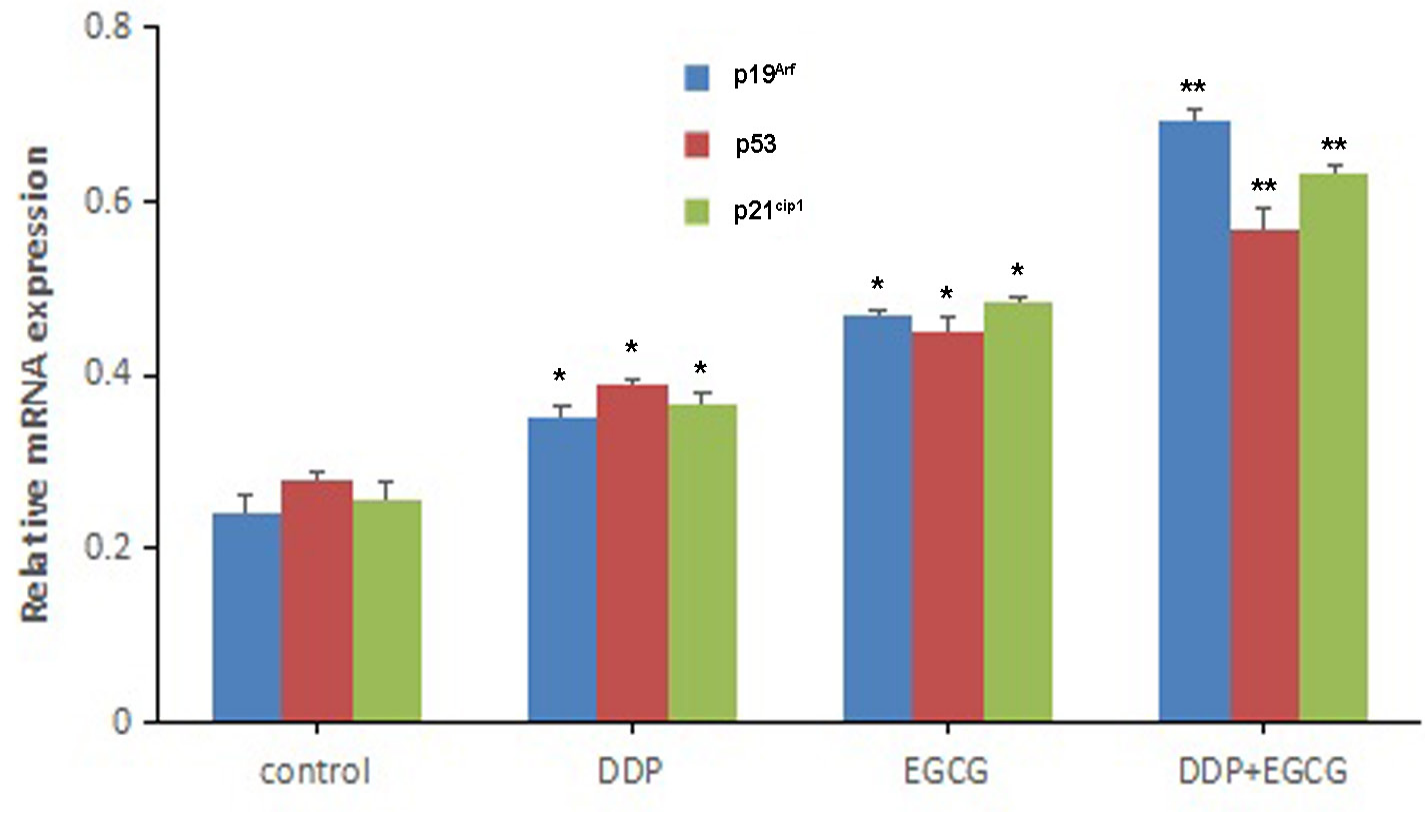
*p19* ^Arf^ *, p53*, and *p21*^Cip1^ Gene Expression was Evaluated by RT-qPCR. Compared with control *P<0.05, **P<0.01

RT-qPCR was used to evaluate *p19*^Arf^*, p53*, and *p21*^Cip1^ gene expression changes. Treatment with EGCG and DDP had positive effect on *p19*^Arf^*, p53 *and *p21*^Cip1 ^mRNA expression levels. Compared to EGCG and DDP treated alone, the combination of EGCG and DDP caused significantly up-regulation of the expression levels of p19^Arf^, p53 and p21^Cip1^ mRNA (Figure 6).

**Figure 7:**
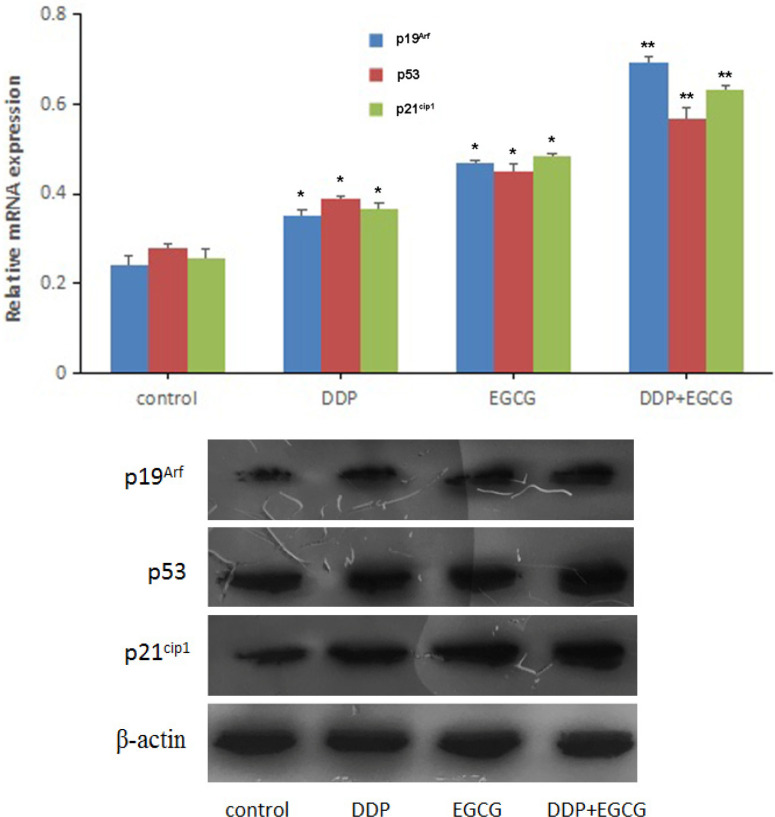
Western Blot was Used to Analyze p19^Arf^, p53, and p21^Cip1 ^Protein Expression. Compared with control *P<0.05, **P<0.01

As shown in Figure 7, the protein expression levels of p19^Arf^, p53 and p21^Cip1^ were relatively low in the control-treated BGC-823 cells. The protein expression of p19^Arf,^ *p53* and *p21*^Cip1^ were activated in treatment of the cells with co-treatment with EGCG and DDP or individual treatments. Following treatment of the cells with the combination of EGCG and DDP, the levels of p19^Arf^, p53 and p21^Cip1^ protein expression significantly increased than that in cells treated with EGCG or DDP alone.

**Table 1:** Primers were Used in RT-qPCR

Primer	Sequence	Annealing Temp (^o^C)
*p19* ^Arf^ *-F*	5'-CCATGTGGACCTGTCACTGT-3'	60
*p19* ^Arf^ *-R*	5'-AAGATGTAGAGCGGGCCTTT-3'	60
*p53-F*	5'-CTGGGGAGTCTTGAGGGACC-3'	59
*p53-R*	5'-CAGGTTGTCTAAATTCCTAG-3'	59
*p21* ^Cip1^ *-F*	5'-CTTGTGGAGCCGGAGCT-3'	58
*p21* ^Cip1^ *-R*	5'-TGGTGTCTCGGTGACAAAGT-3'	58
*β-actin-F*	5'-AAAGACCT-GTACGCCAACAC-3'	55
*β-actin-R*	5'-GTCATACTCCTGCTTGCTGAT-3'	55

**Table 2:** The Cloning Efficiency of BGC-823 Cells Treated with EGCG and DDP

Groups	n	The Cloning Efficiency (%)
Control	3	100 ± 4.02
DDP	3	95.00 ± 2.86*
EGCG	3	88.75 ± 3.90*
DDP+ EGCG	3	75.00 ± 3.75**

Compared with control * P<0.05, **P<0.01

## Discussion

Surgery is currently a radical treatment for patients with early gastric cancer, but the efficacy of surgery is often impaired in advanced gastric cancer. In particular, chemotherapy is most popular strategy for patients with cancerous metastases. However, traditional chemotherapeutic drugs in malignant tumor patients were associated with side effects and minimal efficacy, and most effective concentration of chemotherapeutic drugs were near to patients’ maximally tolerated dose. Therefore, it is extremely important to investigate novel treatments to enhance the effect of gastric cancer treatment and minimize adverse advents.

As the major component of green tea, EGCG has been demonstrated to enhance the activity of chemotherapy drugs and with almost no toxic and side effects (Fang et al., 2019). DDP is a heavy metal drug which is widely used as a first-line anti-tumor drug, however, the toxic effects of DDP have been widely reported in the kidney, nervous system, and pancreas (Yin et al., 2020). In this study, we investigated the effects of EGCG and low-dose DDP in BGC-823 cells. The results of DAPI staining assay showed the nuclear staining of cells treated with EGCG and DDP were brighter and deeper and the cellular nuclei became concentrated, the apoptotic cell ratio in the combination of EGCG and DDP was significantly increased comparing with individual treatments. MTT method and cell cloning experiments confirmed that EGCG and DDP can effectively inhibit the proliferation of BGC-823 cells, and the combination of EGCG and DDP enhanced the treatment efficacy. As Zhao (2012) reported in his previous work also demonstrated similar efficacy of EGCG combined with DDP in small cell lung cancer H446 cells. The results of the cell scratch test revealed that co-treatment with EGCG and DDP or individual treatments inhibited cell migration, and the combination of EGCG and DDP exhibited significant inhibitory effect on BGC-823 cell migration ability. Flow cytometry results showed that the early apoptosis cell population in co-treatment with EGCG and DDP significantly increased compared with DDP treated alone, and the population of cells accumulated in G1 phase in the combination of EGCG and DDP was more than individual treatments. The trend of increase in medium is the largest, and the cells population in S phase or G2 phase significantly decreased in co-treatment with EGCG and DDP compared to the single-drug treated cells, indicating that EGCG not only promoted cell apoptosis, but also enhance the sensitivity of DDP chemotherapy treatment efficacy. EGCG enhanced the inhibition of DDP in the proliferation of gastric cancer BGC-823 cells. Therefore, DDP combined with drugs were applicated in clinical patients and reduced the cytotoxicity of DDP with the same efficacy targeted therapy to tumors.

Previous studies have revealed that the p19^Arf^-p53-p21^Cip1^ signaling pathway played an important role in the regulation of cell proliferation. p19^Arf^ was demonstrated to bind to MDM2 and inhibited its activity, induced the expression of p21^Cip1^, affected the cell cycle process, and blocked cells in G1 stage and caused cell apoptosis through activating p53, and increasing the expression of p53 (Molofskyet al., 2005; Llanos et al., 2001). As Zhu (2016) reported in his previous work revealed the tumor suppressor genes, p19^Arf^, p53, and p21^Cip1^ interacted with each other to regulate the cell cycle and inhibited tumor cell proliferation, invasion and metastasis. In our previous study, various concentrations of EGCG were added to KG-1 and THP-1 cells and the results suggested that EGCG up-regulated the expression of p19^Arf^, p53, p21^Cip1^ mRNA and protein, and inhibited cell growth and induced apoptosis. Therefore, these results confirmed that EGCG induced leukemia cell apoptosis via p19^Arf^-p53-p21^Cip1^signaling pathway.

In this study, the expression of mRNA and protein were detected by RT-qPCR and Western blot, and the BGC-823 cells were found to exhibit high expression levels of *p19*^Arf^*, p53, p21*^Cip1^ mRNA and protein in co-treatment with EGCG and DDP or individual treatments. The combination of EGCG and DDP significantly increased *p19*^Arf^*, p53, p21*^Cip1^ mRNA and protein compared with the EGCG or DDP treated alone. These results suggested that the effects of EGCG and DDP on the proliferation, apoptosis and invasion of gastric cancer cells were closely related to the p19^Arf^-p53-p21^Cip1^ signaling pathway. The present study revealed for the first time that EGCG enhanced the inhibition of proliferation, invasion of DDP in gastric cancer cells, and promoted the cell apoptosis through up-regulating the expression of p19^Arf^, p53, p21^Cip1^ via the p19^Arf^-p53-p21^Cip1^ signaling pathway.

In summary, the findings of the present study help illustrate both EGCG and DDP can inhibit the proliferation of gastric cancer BGC-823 cells to achieve a therapeutic efficacy, and the inhibitory effect of co-treatment with EGCG and DDP is better than individual treatments. Furthermore, EGCG enhanced the inhibition of proliferation, invasion of DDP in BGC-823 cells by regulating the p19^Arf^-p53-p21^Cip1^ signaling pathway and the cell apoptosis induction of DDP in BGC-823 cells. However, further confirmation with the combination of EGCG and DDP used in vivo is needed and the underlying mechanisms are required to be elucidated.

## Author Contribution Statement

M.W. and J. L. conceived and designed the research. X. L., B. C. and M. X. performed the experiments. X. L. and X. R. analyzed the data. M. X. and J. L. wrote the manuscript.
